# Book Notices

**Published:** 1868-12-01

**Authors:** 


### *BOOK NOTICES.*

Outlines of Physiology, Human and Comparative. By John Marshall, F.R.S., Professor of Surgery in the University College Hospital; with additions by Francis G. Smith, M.D., Professor of Institutes of Medicine in the University of Pennsylvania. Henry C. Lea, Philadelphia. 1868.

Time and space alike forbid the indulgence of the desire to give an extended notice of this excellent treatise on physiology. Still, however, we can not refrain from saying a few words in commendation of a work which we believe will become popular, among students at least, so soon as they shall have become familiar with some of its merits.

Unlike most book-makers, the author of this volume seems to have been troubled by no lack of subject-matter wherewith to make up a saleable article for the book market. His difficulties have been of quite another character, and the magnitude of his subject, and not less the abundant resources at his command, have tempted him to exceed by far the limits of an unpretending treatise as originally designed, and give to the profession a work which is destined to take high rank amongst professional classics. The book is not the less attractive in that it presents considerable variation from the stereotyped plan of most of the physiological treatises which are at present accessible to the student and to the general reader, and some of these variations in the mode of arrangement and presentation of the subjects, while they may meet the criticism of the hypercritical, will, at the same time, secure the commendation of the tyro who will be grateful to the author who has given him so extensive a view of the science, upon his knowledge of which must mainly rest his hopes of attaining practical accuracy in his professional career. While, as has been already stated, the author has drawn largely upon the stores of physiological science accumulated by preceding and cotemporary observers, he has been by no means only a compiler, but gives evidence of laborious, patient, and intelligent investigation, and careful classification and generalization.

The work is abundantly illustrated with wood cuts, taken from the most reliable authorities, many of them being designed from the author's own observations, and are truthful and well executed. We are glad to perceive that the author arrays himself against the theory of spontaneous generation, which has recently, in France, received a new impetus from the fallacious experiments and sophistical arguments of Pouchet and others: the axiom "*omne vivum ex ovo*," is as clearly unassailable to-day as when it was proclaimed by the great father of medicine two thousand years ago. Of reproduction the author has assumed the division of Carpenter and the older physiologists, into two kinds, sexual and non-sexual. This decision is certainly based upon incomplete analysis and partial comparison of the phases of the reproductive process in different organisms, and must eventually be abandoned when comparisons have been made between these processes in their totalities. Germination in the plant, or fissiparous or gemmiparous reproduction in the lower animals, has apparently little in common with the fertilization of the ovum in the human organism, and if these two phenomena should be regarded as the corresponding phases in the reproductive processes of the classes of organisms referred to, the processes might with propriety, indeed ought to be placed in different categories. But when the phenomena of gemmation or effission respectively are compared with those accompanying the phase of placental separation in the reproductive process of the higher mammalia, the identity of phenomena is so striking as to necessitate the conclusion that these are corresponding phases, and that other correspondencies will be recognized when a more accurate parallelism shall be maintained in the comparison of reproductive processes throughout the entire organic world.

We would here take exception to the total silence maintained by our author, in common with nearly every other physiologist regarding the influence of moral and physical agencies in the modification of physical function and structure. Man is considered exclusively as a material organism, subject to the operations of material agencies, either external or internal, solely, or at the furthest, to those of a purely emotional character. In this there is an apparent incompleteness in the analysis of functional correlations; for, while the most careful and laborious research has been expended in the investigation of, and due consideration given to the relations of the lower portions of the nervous system with the purely vegetative and animal functions of the organism, and to a certain extent those of the emotional centre, the mesocephale with the same have also been considered. The still more important relations of this latter portion of the nervous system, with its dominant organ, the cerebral hemispheres, have been tacitly abandoned by the physician to the metaphysician as some thing truly *μετα τα φυϐικα*, and those of the organ of intellection and volition, with all the rest of the organism over which it is theoretically admitted to dominate, is totally ignored. This disregard of the psychical relations of the human organism must, by excluding the flood of light which might be thrown thence upon the more subtle and intricate modifications of function and structure, both in health and disease, which now so completely baffle the investigator, must prove an effectual barrier to the progress, not only of physiological science, but of therapeutical efficiency.

We demand for physiology a more extensive scope, a more comprehensive insight into man's nature, inseparably three-fold, and insist that in restricting his investigations to the purely physical and material aspects of the subject, and the abandonment of the moral and intellectual to the theologian and the metaphysician exclusively, the physiologist has relinquished the richest portions of his rightful domain.

Of the typographical and mechanical work it is superfluous to say any thing more in commendation than that it comes from the press of H. C. Lea, of Philadelphia, whose name alone is a sufficient guarantee of excellence in this department. W. H.

---

Recherches' experimental sur une fonction du foie consistant dans la séperation de la cholestérine du sang et son élimination sous forme de stercorine (Seroline de Boudet) par Austin Flint, fils, Doctor en Médicine, Professeur de physiologie et de microscopie au College de Médicine de Bellevue Hospital à New York, et au College de Long Island, Hospital à Brooklyn ; membre de l' academie de Médicine de New York, etc. Paris: Germer Balliere, Libraire Editeur. New York: D. Appleton & Co., 1868.

We are indebted to the courtesy of Messrs. Appleton & Co. for the valuable treatise whose title is prefixed, a familiar friend, not the less welcome that it comes to us clothed in a foreign garb. The appearance of this work in its new guise is a double source of congratulation ; first, to its author, that he has received so flattering a recognition, which he so richly merits, in what he himself terms "*le foyer de la science physiologique*," and secondly, to that "focus" itself in that it has acquired an additional ray of scientific light to aid in maintaining its perennial brightness. It is not inappropriate too, that our young American physiologist should, in this French edition of his work, make to the physiology of France a testimonial of acknowledgment of the source from which he undoubtedly derived his scientific facts, not less than his inspiration to the labor of investigation to which we are indebted for such solid results.

### FLINT ON CHOLESTERINE.

The author has commenced his investigation into the subject cholesterine, *ab initio*, by a thorough analysis of the literature of the subject, from its discovery by Poulletier de la Salle, in 1782, to the present day, starting with the justifiable assumption of Robin et Werdeil, that the physiological role which it fills in the economy, is unknown; he is led to the hypothesis of its analogy, both physiological and pathological, with urea; maintaining that the discovery of cholesterine will effect as much toward the elucidation of many obscure maladies which may be hereafter classified under the title cholesteræmia, as the discovery of urea has effected for those now recognized under the title of uræmia.

The author's experiments upon bile, performed upon dogs, furnish an interesting explanation of the discrepancies perceptible between those of Bidder and Schmidt and Schwan, and those of Blondlot, and at the same time establish the possibility of the restoration of continuity, once interrupted, in the excretory ducts of glands, and likewise determine a new function for the bile, viz.: the separation of cholesterine from the blood. In pursuing his investigations further, he traces this excrementitious substance, cholesterine, from its formation by the disintegration of tissue and its transportation in the current of the circulation to its separation from the blood by means of the liver, and also the disturbances produced in the economy by its retention in the blood, in consequence of failure in the excretory activity of the liver, which condition he very appropriately designates cholesteræmia. Regarding the physiological relations of cholesterine and stercorine, the author has demonstrated the four following propositions:

1st. Cholesterine is an excrementitial matter, produced by the disassimilation of nerve substance, and absorbed by the blood.

2nd. It is separated from the blood, during its passage through the liver, enters into the composition of the bile, to which it gives its excrementitious character.

3rd. It is poured out with the bile into the upper portion of the small intestine, where the digestive process changes it into stercorine, under which form it is enveloped in the fæces.*

Apropos of the pathological relations of these two bodies, our author concludes,

1st. That the proportion of cholesterine in the blood is enormously increased, which shows that an organic change in the liver has prevented its separation from this fluid.

2nd. A corresponding diminution of stercorine in the fæces shows that the cholesterine is not poured into the alimentary canal in its normal quantity.

4th. Stercorine, which constitutes the great excrementitious element in the fæces, is one of the most important excrements resulting from the usage of the economy.

It is impossible, within the narrow limits at our command for the purpose, to do full justice to Dr. Flint's monograph; it must be read, yes, studied, to be appreciated, and should be studied, and carefully studied, by every one pretending to physiological knowledge, or practical excellence.

W. H.

---

We have received the third number, for October, 1868, of the *Revista Medico Quinorgica y Dentistica.* Periōdico de las Ciencias Médicas En Todas Sus Ramos, Y. Eco De La

* The discovery, in February, 1868, by the writer, of cholesterine in the fæces of a patient in whom disintegration of the mucous surface of the duodenum had been already established by concomitant symptoms, furnishes confirmation of the proposition. W. H.

Brensa, Médico Estranjira. Bedactores Y. Boprutarious, Los Sres: Wilson Y. Gonzales, Habana, Cuba.

We are most happy to extend a hearty welcome to this youthful stranger from the Queen of the Antilles, and to accede to the request of its editors "to be placed upon our exchange list." Under any circumstances we should receive this journal with pleasure upon its own merits solely, but our pleasure is enhanced by so substantial evidence of the status and *esprít de corps* of our professional brethren in the West Indias. "*La Revista*" is a quarterly of upwards of one hundred pages per number, containing several original articles of a high order of merit, and also admirable selections from the best American and European journals. Many of the articles are handsomely illustrated, and altogether the journal presents an appearance highly creditable to its editors and its publishers.

We shall take the liberty, from time to time, to translate from its pages such items as may appear likely to interest our own readers, as "a fair exchange is no robbery." W. H.

---

ALBUMINURIA: Translated from La Revista Medico Quirurgica, Habana.

Professor Semola, of Naples, announces the opinion that the passage of albumen into the urine in the disease called "Bright's," is a necessary consequence of a general deficiency of nutrition, in virtue of which the albumen, becoming incapable of exercising its functions, is eliminated by the kidneys as a substance foreign to the organism. According to this theory, the alteration of these organs occupies only a secondary part in the pathology of the disease; and yet the condition of the kidneys is an important *datum* in the prognosis. Professor Semola protests against the ideas of those who pretend to explain or to solve the question by virtue of mere anatomical deductions. One of the diagnostic signs which distinguish organic from symptomatic albuminuria, rests upon the quantity of urea, which in true Bright's disease, diminishes from the first appearance of the albumen, and at a later period accumulates in the blood. The same occurs with the sulphates. Of artificial (?) albuminurias, that which originates from suppression of the cutaneous functions, most closely assimilates to Bright's disease. This suppression impedes, on the one hand, the oxidation of substances introduced into the system under the form of peptones, (products of the digestion of albumen), and, on the other hand, occasions congestion of the viscera, especially of the kidneys. Thus, according to this author, Bright's disease is not the result of a primitive anatomical lesion, but the result of that double series of effects which succeeds more or less quickly the suppression of the function of the skin. Consequently, the efforts of the physician ought to be directed to the re-establishment of these functions, by augmenting the oxydation of the peptones and effecting the disappearance of the renal congestion. Amongst the remedies best adapted to this object are to be reckoned ordinary sudorifics, or in obstinate cases, bottles of hot air,* followed always by cool or cold shower baths, the preparations of arsenic, and inhalations of oxygen. The diet ought to be feculent or vegetable, with very little meat.

---

Results of Surgical Operations in Portugal.—In examining the work of Dr. Antonio Maria Barbosa, published in *la Gaceta Médica de Lisboa*, we find that the results of special operations performed by Portugese and French surgeons in Paris and Lisbon, are less favorable than those obtained by the surgeons of London. In the operations for hernia and lithotomy by Dr. Barbosa, we perceive that the advantage is clearly on the side of the Engli h surgeons. Among the former the mortality was as follows: Hospitals of Paris, 60.45 per cent.; in Lisbon, 58.82 per cent.; in London, 50.75 per cent. Among lithotomy operations, the mortality was 37.3 per cent.; in the hospitals of Paris, 35.7 per cent.; and 21.5 per cent. in London.—*Gazeta Medica de Bahia.*

* The Professor does not state how the "bottles of hot air" must be applied or used.

PROF. TURK.—The heirs of this distinguished physician have donated his library, comprising more than a thousand volumes, amongst which are many works of great merit, to the Royal Society of Physicians of Vienna. The instruments for laryngoscopy were assigned to the Surgical Clinic of Professor Dumrischen, and the duplicates to the department of the hospital which was in the charge of the deceased.—*Allgemeine Wiener Medical Zeitung.*

---

PROFESSOR F. VERDUGO died recently in Salamanca, Spain, at the advanced age of 105 years, after having practiced medicine during eighty years.—*Revista Medico Quirurgica, Habana.*

